# Sudden cardiac death during anesthesia in non-cardiac surgery and its link to possible cardiac channelopathies: A case series

**DOI:** 10.1016/j.jcadva.2024.100083

**Published:** 2025-02

**Authors:** Samadhi Dandeniya Arachchi, Joseph Westaby, Mary N. Sheppard

**Affiliations:** CRY Cardiovascular Pathology Unit, Cardiovascular and Genomics Research Institute, St. George's, University of London, United Kingdom

**Keywords:** Sudden cardiac death, Channelopathies, Perioperative cardiac arrest

## Abstract

Cardiac arrest with induction of anesthesia in those who appear healthy with no apparent cardiovascular disease is rare and the autopsy is essential to identify any underlying cardiac pathology. A retrospective analysis of the database of sudden cardiac deaths referred to the CRY Centre, St. Georges University of London identified 7 cases in which death occurred during anesthesia. We found 3 cases with explained causes; peripartum cardiomyopathy, myocarditis and ischemic heart disease where the risk of sudden cardiac death in anesthesia is well known and helps anesthetists and surgeons explain the cardiac arrest. More importantly there were 4 cases with morphologically normal hearts with no apparent cardiovascular disease who developed bradycardia and cardiac arrest with the induction of anesthesia. This raises the possibility of cardiac ion channelopathies when nothing else is found at autopsy and there is no aesthetic cause. The negative autopsy has major implications for the families requiring detailed cardiovascular screening of the immediate family to prevent future sudden deaths. All surgical teams need to be aware of this and advise the families accordingly in these traumatic situations.

## 1 Introduction

The risk of sudden cardiac arrest (SCA) followed by death during anesthesia is a rare, but catastrophic event for families, anesthesiologists and surgical teams [[1], [2], [3], [4]]. Its causes are multifactorial, but it is usually due to an aesthetic and surgical complications or underlying cardiovascular disease including ischemic heart disease. Cardiac arrest with induction of anesthesia in those who are healthy is rare. Autopsy is essential to identify the cause.

We present a series of cases with sudden cardiac death (SCD) reported with anesthesia.

We carried out a retrospective analysis in the national database of SCA during anesthesia resulting in death referred the CRY Centre, St. Georges University of London and identified 7 cases in which death occurred during anesthesia. All cases underwent a full autopsy with no alternative cause of death found. Toxicology and mast cell tryptase to exclude anaphylaxis or other anesthesia related causes. All hearts were examined macroscopically and microscopically by two expert cardiac pathologists as previously described [5,6].

## 2 Case series description

The study has IRB approval by the London Stanmore committee (10/H0724/38).

Details of 7 cases of SCA during anesthesia resulting in death are summarized in Table 1.

**Table 1:** Summary of the cases referred to the CRY Centre between 2013 and 2023

Age [Y]	Gender F/M	Circumstances	Cardiac diagnosis
47	F	Died during induction of GA for removal of retain products postpartum Bradycardia followed by cardiac arrest	Morphologically normal heart
80	F	SA for knee surgery Cardiac arrest during induction of anesthesia with bradycardia & cardiac arrest. Died 3 days later	Morphologically normal heart
55	M	LA given for vitrectomy, At the end of the procedure, he became bradycardic with cardiac arrest. Died 10 days later	Morphologically normal heart
48	F	GA given for laparoscopic cholecystectomy During induction of anesthesia bradycardia and cardiac arrest Died within 24 h	Morphologically normal heart
19	F	SA given for C-section. During induction bradycardia followed by cardiac arrest Died 3 h later	Peripartum cardiomyopathy
89	M	GA given for emergency laparotomy for obstructed inguinal hernia. During induction, Bradycardia with cardiac arrest, Died 2 days later	Chronic Ischemic Heart Disease
13	F	GA for exploratory laparotomy to exclude appendicitis. Bradycardia with cardiac arrest during induction of anesthesia died within 90 min	Myocarditis

[GA-General anesthesia, SA-Spinal anesthesia, LA-Local anesthesia].

In four of the seven cases the autopsy was negative with a morphologically normal heart while in three cases a cardiac diagnosis was made of cardiomyopathy, ischemic heart disease and myocarditis.

## 3 Conclusions

Meticulous autopsy evaluation of all sudden deaths during anesthesia is essential. It gives an insight into cardiac causes of peri-operative mortality to the surgical team and the anesthetists who are usually traumatized psychologically by the death with subsequent investigations and audit. This can help tremendously in explaining the death to the family.

The incidence of perioperative cardiac arrest in non-cardiac surgeries is rare. It occurs at rate of between 0.5 and 1 case per 10,000 cases [1] in adults and has a higher incident in the pediatric age group above 1 per 10,000 cases [7]. Children who are ill, younger (specially neonates) and with underlying congenital heart disease are at a higher risk [8].

Analysis of 170 adult death cases in the Karolinska Hospital revealed 115 cases were an aesthetic related. The commonest causes were hypoxia due to ventilatory problems, post succinylcholine asystole and post-induction hypotension. Cardiac causes were not mentioned [9].

A Chinese study found 104 cardiac arrests with 34 deaths; 11 of the 104 cardiac arrests were anesthesia related and many cases were elderly with cardiovascular events similar to our elderly case [10]. The non-anesthesia related 77 arrests were not discussed in this paper.

We found 3 cases with explained causes; peripartum cardiomyopathy, myocarditis and ischemic heart disease where the role of autopsy is essential in explaining an underlying cardiac cause which is vital to allow the surgical and an aesthetic team explain the arrest to the family. It also shows that these conditions can be asymptomatic prior to surgery. The risk of SCD in anesthesia is well known in ischemic heart disease, cardiomyopathies and myocarditis.

Four had morphologically normal hearts with no apparent cardiovascular disease and no other cause found at autopsy, on toxicology or mast cell tryptase. No anesthesia related cause of death was identified. Three of them died during the anesthesia and one died a few days later as a late complication of arrest. This raises the possibility of cardiac ion channelopathies which are a diagnosis of exclusion.

Ion channelopathies are a group of rare genetic conditions, affecting the cardiac ion channels: Brugada syndrome, Long Q-T syndrome, short Q-T syndrome, Catecholamine-induced polymorphic ventricular tachyarrhythmia. They are well documented in adults and children with structurally normal hearts [5]. Numerous medications used in anesthesia are recognized to prolonged QT interval [11]. In Brugada syndrome, perioperative medications can trigger arrhythmia and there is an elevated risk of SCD for during surgery [12]. Wolff-Parkinson-White syndrome is a disorder due to accessory electrical pathways in the heart, which can also cause SCD during anesthesia [13]. In the 4 cases with a negative autopsy and morphologically normal heart, the families need investigation for the possibility of cardiac channelopathies as the causes are mainly genetic [5]. Anesthetists can review ECGs to assess for any indication of channelopathy and consult cardiology colleagues if they have concerns.

The incidence of cardiac arrest and SCD in general anesthesia is reported higher than that for spinal anesthesia [14]. This is associated with increase vagal activity due to medication routinely used in general anesthesia like propofol and succinylcholine [1,13]. These drugs are reported to cause bradycardia and asystole followed by SCD [15]. The 4 cases in our series which had normal hearts, showed bradycardia and cardiac arrest during induction of anesthesia and one at the end of the procedure. There were 2 of general and 2 of local anesthesia. Vagal response, hypoxia and hypovolemia are some of the mechanisms which can trigger cardiac arrhythmia and arrest during anesthesia which most likely can increase the risk in those with cardiac channelopathies [12]. The negative autopsy cases especially have major implications for the families requiring detailed cardiovascular screening and genetic testing of the immediate family to prevent future sudden deaths.

Autopsy with full cardiac pathological examination is the gold standard for determination of presence or absence of any structural abnormality of the heart in the evaluation of a sudden death during anesthesia. Our series of 7 cases with SCD during anesthesia in non-cardiac surgeries show cardiac disease in 3 cases which explained the death and 4 with negative autopsy findings. These autopsies with detailed cardiac pathology examination helped the surgical team and the family come to terms with the death. All anesthetists and surgical teams need to be aware of this and advise the families accordingly in these traumatic situations.

There are some limitations to the study. We do not have access to the details of drugs given during anesthesia. Family screening and post mortem genetic testing will be performed as part of a future study.

## Funding

CRY unit and MNS are funded by UK based charity Cardiac Risk in the Young.

JW is funded by the National Institute for Health and Care Research (NIHR).

## CRediT authorship contribution statement

**Samadhi Dandeniya Arachchi:** Writing – original draft, Formal analysis, Data curation. **Joseph Westaby:** Writing – review & editing, Validation, Supervision, Formal analysis, Data curation, Conceptualization. **Mary N. Sheppard:** Writing – review & editing, Validation, Conceptualization.

## Declaration of competing interest

None.

## Data Availability

The data that has been used is confidential.
